# Reframing Aging Through AI-Generated Patient Simulations in Pre-Medical Education

**DOI:** 10.1093/geroni/igaf122.409

**Published:** 2025-12-31

**Authors:** Erta Cenko, Neha Rani

**Affiliations:** University of Florida, Gainesville, Florida, United States; University of Florida, Gainesville, Florida, United States

## Abstract

As future healthcare providers, pre-medical students’ perceptions of aging influence how they will interact with older patients. However, misconceptions and implicit biases about aging persist in medical education, often reinforcing stereotypes. This study explores the use of AI-generated videos as an innovative educational tool to help undergraduate health sciences and pre-medical students reframe their understanding of aging. The study follows a three-phase design. First, students complete a survey assessing their baseline perceptions of aging and older adults. Next, they engage with AI-generated video simulations that depict aging patients with diverse health conditions and life experiences. Afterwards, students complete a follow-up survey to measure shifts in their attitudes. In the final phase, students upload their own photos into an AI system that generates “aged” versions of themselves. These personalized avatars are then integrated into additional patient simulations, allowing students to see themselves as older individuals and reflect on their potential aging experiences. By integrating AI-driven experiential learning, this project aims to increase students’ empathy and challenge ageist assumptions in medical education. Preliminary findings will explore changes in students’ perspectives before and after the intervention, highlighting AI’s potential to transform gerontology education in the medical sciences. This approach supports gerontological education across fields, helping create a more age-inclusive healthcare workforce.

